# The visible touch: *in planta* visualization of protein-protein interactions by fluorophore-based methods

**DOI:** 10.1186/1746-4811-2-12

**Published:** 2006-06-26

**Authors:** Riyaz A Bhat, Thomas Lahaye, Ralph Panstruga

**Affiliations:** 1 Department of Plant-Microbe Interactions, Max-Planck-Institute for Plant Breeding Research, Carl-von-Linné-Weg 10, D-50829 Köln, Germany; 2 Institute of Genetics, Martin-Luther University, Weinbergweg 10, D-06099 Halle, Germany

## Abstract

Non-invasive fluorophore-based protein interaction assays like fluorescence resonance energy transfer (FRET) and bimolecular fluorescence complementation (BiFC, also referred to as "split YFP") have been proven invaluable tools to study protein-protein interactions in living cells. Both methods are now frequently used in the plant sciences and are likely to develop into standard techniques for the identification, verification and in-depth analysis of polypeptide interactions. In this review, we address the individual strengths and weaknesses of both approaches and provide an outlook about new directions and possible future developments for both techniques.

## Background

Having the first completed plant genomes of the monocotyledonous and dicotyledonous reference species rice (*Oryza sativa*) and thale cress (*Arabidopsis thaliana*) in hand [1-3], the analysis of protein function(s) represents a major scientific challenge of the post-genomic era. Many researchers who identified key components of various biological processes by forward or reverse genetic approaches in the past now face a possibly harder task to assign (a) biochemical role(s) to their favorite protein(s). State-of-the-art studies to address this pivotal question frequently involve the analysis of protein-protein interactions to gain insights about the potential cellular function(s) of a protein of interest (POI). Traditionally, the yeast two-hybrid approach represents the method of choice to unravel protein interaction partners of POIs on a large scale and in an unbiased manner [4]. However, since yeast two-hybrid screens are well known to produce false-positive results, subsequent verification of individual interaction partners by further, preferentially *in planta*, approaches is generally desired. Co-immunoprecipiatation ("pull-down"; [5]), *in vitro *association studies (e.g. gel overlay assays or "far Western blots" [6], surface plasmon resonance spectroscopy [7]), blue native gel electrophoresis [8], bioluminescence resonance energy transfer (BRET) [9] and fluorescent protein-based methods [10-12] are nowadays commonly used to achieve this goal. In this review, we focus on the latter, non-invasive, microscopy-based approaches with a particular emphasis on fluorescence resonance energy transfer (FRET) and bi-molecular fluorescence complementation (BiFC) both of which allow monitoring protein-protein interactions *in vivo *and in real time. Though only recently introduced to the plant sciences, both microscopic techniques have been rapidly absorbed by the community of plant scientists. Given the rapid pace of newly emerging fluorophores with ever improved biophysical properties [11], FRET and BiFC are likely to become even more valuable and common tools in the near future.

### Getting started: general considerations for FRET and BiFC studies

Before starting any fluorophore-based *in planta *protein-protein interaction assays, one should take some general considerations into account. First, it should be noted that all fluorophore-based methods require tagged variants of the POIs, modifications that may alter their physiological parameters. Thus, wherever possible, fluorophore-tagged POIs should be tested for *bona fide *subcellular localization, stability, and biological activity. The latter can for example be achieved by complementation of mutant phenotypes or, alternatively, by determining protein activities in *in vitro *assays. Since, conventionally, POIs can be tagged either N- or C-terminally, and since the site of tagging may determine experimental success in an empirical manner, all possible pair wise combinations should be tested when performing FRET or BiFC assays. Unfavorable circumstances, e.g. terminal targeting signals or transmembrane domains, may however preclude some of these theoretically possible combinations. It should be mentioned that, in principle, some POIs might also be tagged internally [13].

A second aspect that needs to be considered is the expression level of the tagged POIs. Frequently, expression is driven by strong constitutive promoters (e.g., the cauliflower mosaic virus [CaMV] 35S promoter) that may result in ectopic expression and/or overexpression. This might subsequently result in artifacts that may possibly either promote or inhibit particular protein-protein interactions. Thus, wherever possible, the native gene promoters should be used for driving the expression of fluorophore-tagged POIs. It should be stressed, however, that due to the method of gene transfer (e.g. particle bombardment, Agroinfiltration) even constructs with own promoters can result in overexpression when multiple gene copies are transferred into single target cells.

The target species and tissue for transgene expression should also be carefully selected. Ideally, the fluorophore-tagged POIs should be expressed in the homologous plant species and in a tissue type that is of biological relevance for the POIs and/or the anticipated protein-protein interaction. Wherever possible, expression should take place in respective (double) null mutants, since endogenous, untagged copies of the POIs may interfere with the protein-protein interaction assay, e.g. by out competing interaction partners. Lines homozygous for T-DNA insertions in the genes encoding both interaction partners represent thus suitable genetic backgrounds for *in planta *interaction assays. Since it is usually difficult to meet all the criteria mentioned above, one should at least attempt to fulfill as many as possible. In the ideal scenario, however, transgenic lines expressing both fluorophore-tagged POIs under control of their own promoters in a respective double mutant genetic background would be used.

Finally, we would like to stress that both FRET and BiFC represent methods that determine "only" the close physical proximity of two fluorophore-tagged fusion proteins *in vivo*. It might be debatable whether such a tight contact is the final proof of a true protein-protein interaction or, alternatively, represents merely an indicator of close vicinity, as for example, the co-localization of two polypeptides in a small plasma membrane microdomain (e.g. [14]) or co-presence of two POIs in a large multi-protein complex. Convincing evidence for a direct as opposed to an indirect interaction currently requires *in vitro *assays using purified recombinant proteins, e.g. the above-mentioned "far Western blots" [6] or surface plasmon resonance spectroscopy [7]. In our view, biologically significant protein-protein interactions are in addition characterized by the involvement of essential amino acid residues in the contact zones of both interaction partners. Mutant variants that are affected in these critical residues and that result in loss of the interaction coincident with an altered plant phenotype are therefore suitable controls to verify the biological significance of a protein-protein interaction. Such mutant variants may originate from genetic screens *in planta*, might be predicted based on educated guesses or structural data, or could be obtained from yeast-based high-throughput loss-of-interaction assays.

### The basic principle of FRET

Förster (or Fluorescence) Resonance Energy Transfer (FRET) is a biophysical phenomenon that was originally discovered more than half a century ago [15]. Its occurrence is based on a long-range dipole-dipole resonance interaction in which non-radiative energy is transferred from a chromophore in an electronic excited state serving as a "donor", to another molecule (fluorescent or otherwise) serving as the "acceptor". This energy transfer leads to a reduction in the donor's fluorescence intensity and a decreased lifetime in the excited state. If the acceptor molecule is likewise a fluorophore, then FRET additionally gets manifested in the form of an increase in the acceptor's emission intensity.

**Figure 1:**
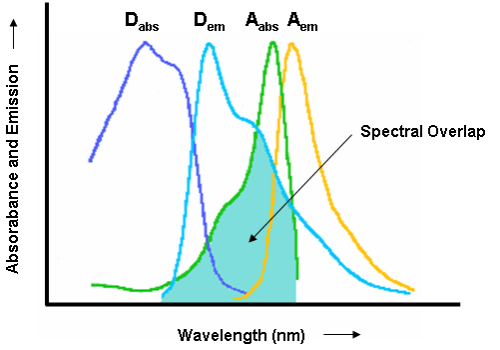
**Excitation and emission spectra of a commonly used FRET pair**. The scheme depicts simplified absorbance and emission spectra of CFP (cyan fluorescent protein; donor; D) and YFP (yellow fluorescent protein; acceptor, A). Overlap between CFP emission and YFP absorption (shaded region) is a prerequisite for FRET. D_abs _– Donor absorbance; D_em _– Donor emission; A_abs _– Acceptor absorbance; A_em _– Acceptor emission.

The efficiency of energy transfer (E) is inversely proportional to the sixth power of the distance between the donor and the acceptor [15,16]:

E = 1/{1 + (R/R_o_)^6^}

where R_o _is the distance at which half of the energy is transferred from the donor to the acceptor. R_0 _is typically between 20–60 A° (2–6 nm) and thus in the range of conventional protein dimensions. The exact value of R_0 _is a function of the spectral overlap between donor emission and acceptor excitation spectra (Figure 1), the quantum yield of the donor in the absence of the acceptor, and the relative orientation and rotational freedom of donor and acceptor chromophore transition dipoles. FRET is unique as it is based on molecular interactions in the 1–10 nm range that are sensitive to molecular conformation, association, and separation and thus represents one of the few tools available for measuring nanometer scale distances or changes in such distances [16,17].

### FRET as a sensor of protein-protein interactions in living cells

**Figure 2:**
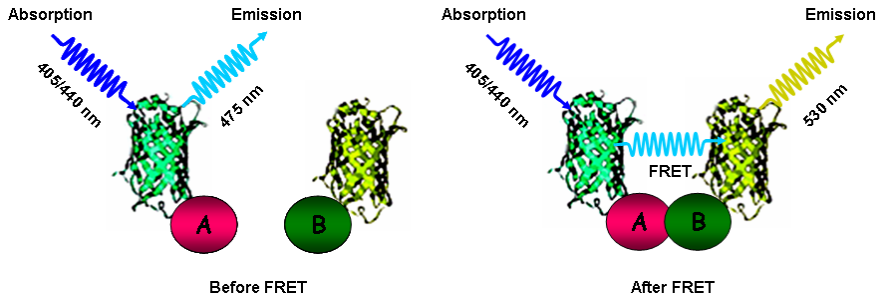
**Detection of protein-protein interactions *via *FRET**. FRET between cyan fluorescent protein (CFP) as a donor fused to protein A and yellow fluorescent protein (YFP) fused as an acceptor to protein B. Under favorable spatial and angular conditions, interaction between A and B causes a decrease in the intensity of donor (CFP) fluorescence concomitant with an increase in acceptor (YFP) fluorescence. CFP and YFP are depicted as cyan and yellow ribbon models fused to putative interacting proteins A and B, respectively.

The availability of genetically encoded fluorophores (green fluorescent protein, GFP; [18,19] and subsequent development of GFP derivatives with suitable spectral properties for FRET (described in [20]) enabled the convenient employment of the FRET principle to address questions in biological systems. In living cells, FRET can occur when protein domains fused to suitable donor and acceptor fluorescent dyes physically interact, i.e. the fluorophores come in close spatial proximity ([10,12]; Figure 2). Such interactions, e.g. between protein domains, can either occur intermolecularly or intramolecularly. Hence, FRET can principally be used to detect either bimolecular protein-protein interactions or conformational alterations within a single polypeptide. In the case of studying an intermolecular interaction, two separate fusion proteins – one containing a donor fluorophore and the other, its putative interacting partner, containing an acceptor fluorophore – are co-expressed in the cell type of choice. If intermolecular FRET is detected this provides direct proof of close proximity of the two chromophores and consequently evidence for the existence of the protein-protein interaction (Figure 2). Alternatively, for analysis of an intramolecular interaction, fluorophores are fused to different sites (frequently the termini) within a single polypeptide. In this case, relative changes in the FRET intensity are indicative of conformational changes within the test protein, e.g. due to ligand binding, maturation, proteolytic processing etc.

During the past few years, FRET has been extensively used to study protein-protein interactions in a diverse range of organisms and cell types, including yeast [21], animal (e.g. [22,23]) and plant cells [24-37]. Likewise, intracellular sensors based on intramolecular FRET gained increasing attention and are now routinely used as nanosensors to report various intracellular changes of metabolites, e.g. alterations in calcium levels [38] or carbohydrate concentrations [39]. However, despite the widespread interest in detecting protein-protein interactions using FRET microscopy, in the plant sciences reports of successful FRET are still limited in number (Table 1).

**Table 1:** Examples of plant protein-protein interactions studied *via *FRET or BiFC.

**Protein-protein interaction**	**Applied technique**	**Cell type**	**Gene transfer method**	**reference**
Phytochrome B-Cryptochrome 2	FRET (channel and DFRAP)	Tobacco protoplasts	Protoplast transfection	[24]
SERK-1 (homodimerization)	FRET (FSPIM)	Cowpea mesophyll protoplasts	Protoplast transfection	[25]
Floral binding protein 2 (homodimerization) Floral binding protein 11	FRET (FSPIM and FLIM)	Petunia leaf protoplasts	Protoplast transfection	[26]
TGA5 (homodimerization)	FRET (channel)	Tobacco leaf cells	Agroinfiltration	[27]
SERK1-KAPP	FRET (FSPIM)	Cowpea mesophyll protoplasts	Protoplast transfection	[28]
Opaque2-CGN5/ADA2	FRET (DFRAP and FLIM)	Cowpea mesophyll protoplasts	Protoplast transfection	[29]
Lipidated YFP and CFP variants	FRET (FSPIM and FLIM)	Cowpea protoplasts	Protolplast transfection	[30]
AtMinD1 (homodimerization)	FRET (channel and DFRAP)	Tobacco leaf epidermal cells	Particle bombardment	[31]
MLO-calmodulin	FRET (DFRAP and FLIM)	Barley leaf epidermal cells	Particle bombardment	[32]
MLO (homodimerization)	FRET (DFRAP)	Barley leaf epidermal cells	Particle bombardment	[33]
Vacuolar ATPase subunits	FRET (channel)	Arabidopsis leaf mesophyll protoplasts	Protoplast transfection	[34]
MADS box proteins	FRET (FLIM)	Cowpea and Petunia leaf protoplasts	Protoplast transfection	[35]
SAG101-EDS1	FRET (DFRAP)	Arabidopsis leaf epidermal cells	Particle bombardment	[36]
AtMinE1-AtMinD1, AtFtsZ1-1-AtFtsZ2-1, AtFtsZ2-1-ARC6	FRET (method unknown)	Tobacco leaf epidermal cells	Particle bombardment	[37]
bZIP63 (homodimerization)	BiFC	Tobacco leaf epidermal cells	Agro-infiltration	[68]
LSD1 (homodimerization)	BiFC	Arabidopsis leaf epidermal cells	Agro-infiltration	[68]
14-3-3 (homodimerization)	BiFC	Arabidopsis cell culture protoplasts and tobacco leaf epidermal cells	Protoplast transfection and Agro-infiltration	[68]
PFTα-PFTβ	BiFC	Arabidopsis leaf epidermal cells	Agro-infiltration	[69]
FIE-MEA	BiFC	Tobacco and Arabidopsis leaf epidermal cells	Agro-infiltration	[69]
VIP1-VirE2, VIP1-VirF	BiFC	Tobacco and onion leaf epidermal cells	Particle bombardment	[70]
SAD-GAMYB	BiFC	Onion leaf epidermal cells	Particle bombardment	[71]
OFP1 (homodimerization), BLH1 (homodimerization), AtOFP1-AtOFP1	BiFC	Tobacco leaf cells	Agroinfiltration	[72]
VirE2-VirE3	BiFC	Tobacco and onion leaf epidermal cells	Particle bombardment	[73]
VIP1-VirE2	BiFC	Tobacco leaf epidermal cells	Particle bombardment	[74]
VIP1-H2A	BiFC	Tobacco leaf epidermal cells	Particle bombardment	[75]
EID1-ASK1	BiFC	Mustard seedlings and parsley protoplasts	Particle bombardment and protoplast transfection	[76]
FD-FT	BiFC	Tobacco leaf epidermal cells	Agro-infiltration	[77]
AtMinE1-AtMinD1, AtFtsZ1-1-AtFtsZ2-1, AtFtsZ2-1-ARC6	BiFC	Tobacco leaf epidermal cells	Particle bombardment	[37]
OsOBF1 (homodimerization), OsOBF1-LIP19	BiFC	Onion bulb epidermal cells	Particle bombardment	[78]
ATH1-STM, BLH3-STM, BLH9-STM	BiFC	Leek epidermal cells	Particle bombardment	[79]
p6 and TGBp2 topology	BiFC	Tobacco leaf epidermal cells	Particle bombardment and Agro-infiltration	[80]

Although an inherently extremely inefficient process, recent advances have led to quantitative and qualitative improvements in the FRET technique including increased spatial resolution, distance range and sensitivity [40]. A major problem, however, that remains is achieving FRET in the first instance, because a successful FRET readout requires that the donor and acceptor fluorophores come into close proximity. This can be a limiting factor, especially in the case of large interaction partners (please note that FRET efficiency is inversely correlated with the sixth power of the distance between donor and acceptor fluorophores; see above; [41]). Sterical orientation of the fluorophores in the fusion proteins is another critical and possibly limiting factor [41]. Both fluorophore distance and orientation represent parameters that are difficult to control, except by the empirical insertion of "spacer" sequences between POI and the respective fluorophore. The length and/or amino acid sequence of such spacer sequences have been shown to either positively or negatively influence inter- and intramolecular FRET efficiencies [42-45].

### Measuring FRET: being spoilt for choice

Upon transfer and expression of suitable transgene pairs into target cells, FRET can be measured by several techniques that differ in the precision of data acquisition as well as the complexity of the required instrumentation (online supplement of reference [46]). A decrease in the donor fluorescence intensity (or the quantum yield) and its excited state lifetime, with a corresponding increase in the acceptor fluorescence intensity (if the acceptor is fluorescent) are the photophysical consequences of FRET. Accordingly, methods for measuring FRET and hence intra- or intermolecular interactions rely on assessing one or more of the above photophysical consequences. Documentation of FRET can be either achieved by rather simple methods like channel FRET or fluorescence spectral imaging microscopy (FSPIM), or by advanced technologies like donor fluorescence recovery after photobleaching (DFRAP) or fluorescence lifetime imaging (FLIM).

Conventionally, FRET was determined by comparing the donor intensity of the donor-acceptor sample to that of the donor only sample, while concurrently comparing the acceptor intensity of the donor-acceptor sample to that of the acceptor only sample (e.g., [24,27,31,34]; Table 1). This method, also known as sensitized emission or channel FRET, requires matching (equimolar) concentrations of fluororohores in the different samples which, being dependent on the cellular expression levels of the proteins under study, is difficult to achieve in an accurate manner. Besides, the direct excitation of the acceptor fluorophore at donor excitation wavelengths requires the subtraction of cross talks and false FRET values using several instrumental correction factors [47]. Additionally, for plant cells, it was reported that the chlorophyll pigments might absorb part of the donor fluorescence and thus lead to false FRET values [48]. Though different mathematical corrections of sophisticated complexity have been designed to rectify these problems [47,49,50], this approach has become less popular due to the development of more reliable FRET techniques (see below).

Fluorescence spectral imaging microscopy (FSPIM) represents a different procedure to document FRET. The method uses a spectroscopic rather than an image-based approach to quantify changes in the acceptor intensity at the donor excitation wavelength. This is achieved by recording emission spectra of the acceptor molecule in the absence or presence of the donor. In comparison to channel FRET, this approach is less sensitive to background noise since spectral rather than intensity information is used as readout. However, as in the case of sensitized emission (see above), a prerequisite is the expression of equal (equimolar) concentrations of the fluorophores, a condition that might be difficult to obtain. Despite this obstacle, the FSPIM procedure has been previously used in various plant FRET studies [25,26,28,30] (Table 1).

**Figure 3:**
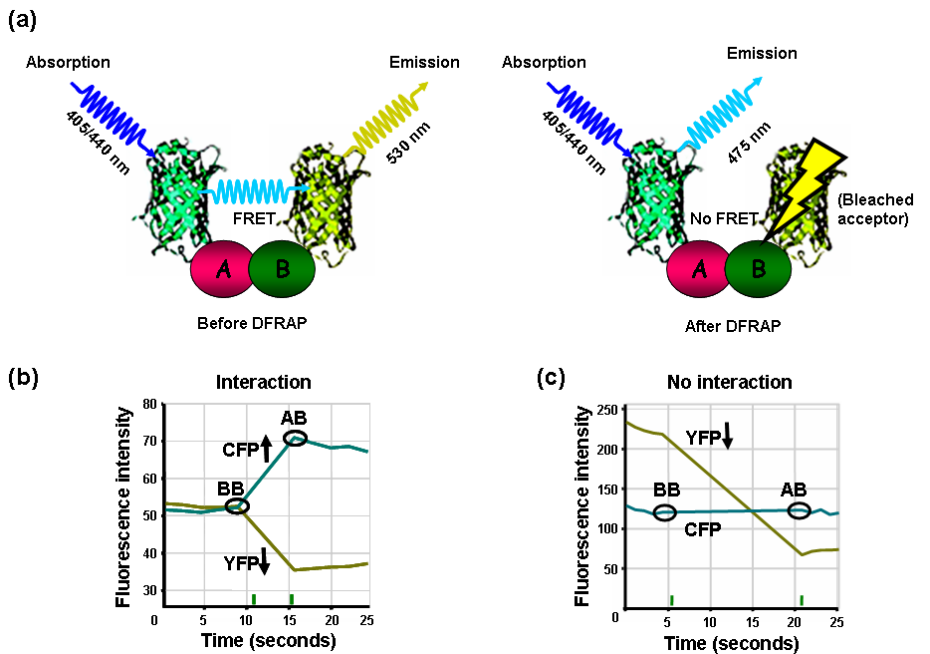
**Principle and quantitative assessment of FRET *via *DFRAP**. **(a) **In case of FRET between the donor CFP and the acceptor YFP due to interaction between two proteins A and B, the photochemical destruction of the acceptor abolishes FRET and leads to an increased emission from the donor, CFP. CFP and YFP are depicted as cyan and yellow ribbon models fused to putative interacting proteins A and B respectively. **(b, c)**. Time-course analysis of fluorescence intensity before and after photobleaching in the presence or absence of a protein-protein interaction. Blue and yellow curves indicate the levels of CFP and YFP fluorescence before and after photobleaching, respectively. In case of FRET, bleaching of the acceptor molecule leads to an increase in donor fluorescence **(b)**. In the absence of interaction between proteins A and B, CFP levels before and after the bleach do not vary considerably **(c)**. BB – Before bleach, AB – After bleach.

A further improvement to measure FRET is DFRAP, donor fluorescence recovery after photobleaching [51]. This procedure is based on the fact that energy transfer from the donor to the acceptor fluorophore will be disrupted upon the irreversible photochemical damage of the acceptor by photobleaching (Figure 3a). As a consequence, the donor fluorescence emission will increase over a short period of time until the acceptor becomes available again (by diffusion from other areas of the live cell) and FRET is re-established (Figure 3b). Since donor fluorescence usually remains unaltered or even decreases after bleaching in the absence of FRET ([51]; Figure 3c), an increase in donor fluorescence is considered a reliable indicator of successful energy transfer. Furthermore, an increase in donor intensity can not be attributed to acceptor bleed through, because the acceptor is not available anymore due to photobleaching. In contrast to channel FRET or FSPIM, DFRAP is also less sensitive to potential artifacts due to unequal expression levels of the fusion proteins [52]. Owing to the fact that the high mobility of some (e.g. cytoplasmic) polypeptides may accelerate the undesired recovery of FRET, DFRAP measurements should be restricted to a narrow time slot of a few seconds following bleaching (Figure 3). In a range of studies this technique was employed to monitor FRET in various plant systems [24,29,31-33,36] (Table 1). A recent report, however, describes that in DFRAP experiments photoconversion of the bleached YFP into a CFP-like species may occur *via *an as yet unknown mechanism [52] – an incident that may affect any DFRAP measurements. Although we could not observe such a phenomenon under our experimental conditions (Bhat and Panstruga, unpublished results), this report raises a genuine concern about the employment of DFRAP as a sole FRET sensor.

**Figure 4:**
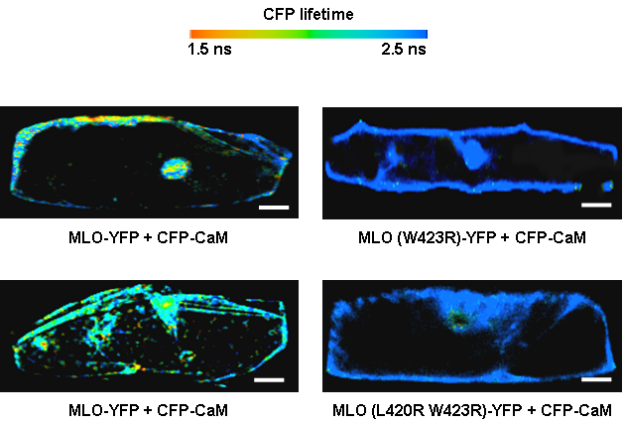
**FRET-FLIM analysis of the MLO-calmodulin interaction**. Barley MLO is a plant-specific calmodulin-binding protein that functions as a modulator of defence against the common powdery mildew pathogen [90]. YFP-tagged wild-type barley MLO or mutant variants thereof (W423R and L420R W423R, bearing amino acid substitutions in the calmodulin binding domain [90]) were co-expressed with CFP-tagged calmodulin in single barley leaf epidermal cells. FRET-FLIM analysis was performed as described in [32]. Donor fluorophore lifetimes are color-coded according to the scale indicated on top of the Figure. "Warmer" colors are indicative of shorter donor fluorophore lifetimes and thus interaction between MLO and calmodulin. Size bar, 20 μm.

Finally, the most sophisticated but also a technically demanding way of determining FRET is by measuring the lifetime (the average time that the fluorophore spends in the excited state) of the donor in the presence and absence of the acceptor. This procedure exploits the biophysical fact that FRET leads to a decrease in the donor life-time that can be determined using suitable equipment. Fluorescence life-time imaging microscopy (FLIM) allows the measurement of changes in fluorophore life-times down to pico-second levels [53,54]. FLIM measurements, as opposed to simple intensity or DFRAP measurements, have the advantage of being concentration-independent and also free of interference by spectral cross-talk, photobleaching or absorption of the donor fluorescence by chlorophyll [26]. The latter can become a problem in FSPIM studies or sensitized emission assays when looking at the quenching of the donor or the sensitized emission of the acceptor, respectively. Additionally, if multiple lifetimes can be resolved, FLIM is able to differentiate subpopulations with different amounts of energy transfer [48] and thus provides a quantitative interaction map of a cell with a single measurement (Figure 4).

Several recent studies have used the potential of FLIM to capture interactions between partner proteins playing different roles in regulation of transcription, development as well as disease signaling in plants [26,29,30,32,35] (Table 1). However, the technique is still far from becoming a routine method for monitoring protein-protein interactions in plants or any other system. Major obstacles are the associated costs and the current limited availability of lifetime systems. Additionally, setting up the FLIM system for determining FRET can be laborious and technically demanding. The commercialization of systems with improved and dedicated image analysis software should increase the popularity of lifetime imaging.

### The choice of fluorophores for FRET

Pairs of fluorophores with overlapping emission and excitation spectra are a prerequisite for FRET (Figure 1). Ideally, the acceptor should exhibit minimal excitation at the wavelength used to excite the donor fluorophore. Chromophore-mutated green fluorescent proteins (GFPs) with an excellent spectral overlap have been widely used in FRET studies [55]. Initially BFP (blue fluorescent protein) was heralded as an ideal FRET partner with GFP [48]. However, owing to the low photostability of BFP [51], identification of the CFP (Cyan fluorescent protein) and YFP (Yellow fluorescent protein) mutant versions of GFP replaced the BFP-GFP pair as donor-acceptor couple in FRET studies. Since then, the original CFP-YFP FRET pairs or their mutant versions such as monomeric mCFP and mYFP, Cerulean (a brighter CFP), Venus and Citrine (both improved YFPs) or recently identified CyPet and YPet are being extensively used for FRET studies in living cells [56-58]. Identification and use of RFP (Red fluorescent protein) as an acceptor to the GFP in FRET experiments was also exploited successfully when Más et al. [24] used GFP-RFP as a donor-acceptor FRET pair to analyse the molecular interaction between Arabidopsis phytoreceptors PHY-B and CRY2. Recently described mutations in RFP to produce fluorescent proteins over the whole visible spectrum, e.g mOrange, mPlum, mCherry etc. [56,59], have opened up the possibility of using these as acceptors with GFPs like T-Sapphire (an improved GFP with a single excitation peak and a huge Stoke's shift; excitation wavelength 399 nm; emission wavelength 511 nm) as donors [60]. Generally, monomeric fluorophore versions should be used [61] to minimize the reported low-affinity oligomerization of GFP variants that may affect FRET measurements [62]. A comprehensive review article about the choice of fluorophores, including FRET studies, has been recently published and is referred to for further details on this topic [56].

### Bimolecular fluorescence complementation (BiFC)

**Figure 5:**
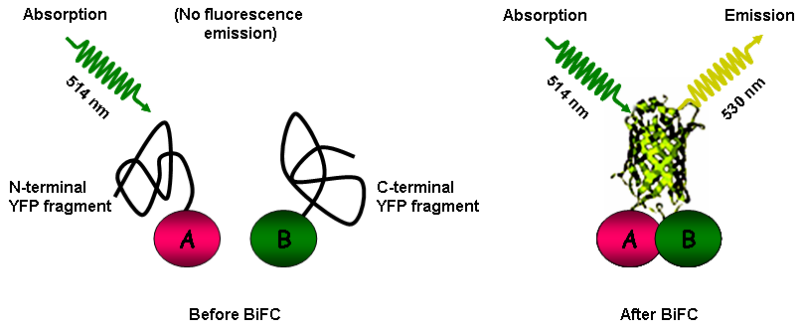
**Principle of the BiFC assay**. The scheme depicts the principle of the BiFC assay, exemplified by a split YFP fluorophore. Proteins A and B are fused to N- and C-terminal fragments of YFP, respectively. In the absence of an interaction between A and B, the fluorophore halves remain non-functional. Following interaction between A and B, a functional fluorophore is reconstituted which exhibits emission of fluorescence upon excitation with an appropriate wavelength.

**Figure 6:**
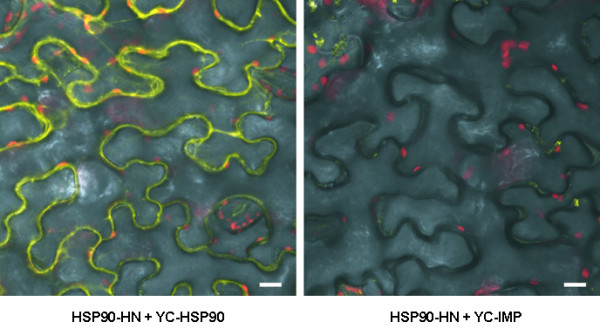
**Confocal images of bimolecular fluorescence complementation (BiFC) studies**. The micrographs show a positive result (HSP90 dimerization; [91]) as well as a negative result (expected absence of interaction between HSP90 and importinα, a mediator of nuclear transportation) of the BiFC assay. HSP90 tagged with the N-terminal fragment of YFP (HSP90-YN) was co-expressed in *Nicotiana benthamiana *leaves by *Agrobacterium tumefaciens *transient transformation with the C-terminal fragment of YFP fused to either HSP90 (YC-HSP90; left side) or importinα (YC-IMP; right side). Yellow colour results from the functional complementation of the two halves of the YFP fluorophore and indicates interaction of corresponding fusion proteins. Size bar, 10 μm.

The BiFC (also known as "split YFP") assay is based on the observation that N- and C-terminal sub fragments of GFP (or derivatives thereof, e.g. YFP) do not spontaneously reconstitute a functional fluorophore. However, if fused to interacting proteins, the two non-functional halves of the fluorophore are brought into tight contact, refold together and generate *de novo *fluorescence. Thus, by BiFC, the interaction status of two POIs can be easily monitored *via *fluorescence emission upon excitation with a suitable wavelength (Figures 5 and 6).

As many other interaction reporter systems, the BiFC assay is a fragment complementation assay (FCA). GFP and its variants are especially attractive reporters for FCA-based interaction studies, because i) no exogenous reagent needs to be added to detect the reassembled protein and ii) GFP and its derivatives are known to express, fold, mature and fluoresce in virtually every cell type and subcellular structure in which they have been tested. Mutational studies uncovered permissive sites within the GFP molecule that allow insertions without disturbing GFP fluorescence [63,64] and thus paved the way to create a GFP-based FCA assay. Subsequently many different split points have been studied for GFP and its derivatives (reviewed in [65]). Ghosh and colleagues [66] were the first to report the use of a split GFP reporter *in vitro *and in *E. coli *to study protein-protein interaction. Subsequently, Hu and co-workers showed that a split fluorophore reporter can also be used in mammalian cells [67]. Finally, Bracha-Dori and colleagues as well as Walter et al. reported on usage of the BiFC system in plant cells [68,69]. Meanwhile many more reports on the use of BiFC *in planta *have been published [70-80] and the system is becoming a routinely used approach to study protein-protein interactions in living plant cells.

When using BiFC for interaction studies one should be aware of the pitfalls and limitations of this technique. One major drawback as well as an advantage of the BiFC approach lies in the irreversibility of complex formation [81]. This obscures the physiological time course of interactions but also traps and accumulates transient and weak associations, thus increasing the sensitivity of detection. An important question in BiFC studies is if the affinity of two interaction partners corresponds to the degree of cellular fluorescence. A recent study shows that BiFC-derived fluorescence does not directly relate to the protein-ligand dissociation constant for an arbitrary protein-ligand pair [81]. However, when studying several interactors for a given protein, BiFC is indeed useful for discriminating strongly bound ligands from weakly bound ones [81]. A further aspect that needs to be considered is the maturation time of the fluorophore tag. Intact (full-size) GFP, for example, requires several hours to mature in the cell [20], and it is conceivable that the intermolecular reconstitution of a split fluorophore may take even longer. Thus, proteins with high turnover rates might not be amenable to BiFC studies. However, modified fast-maturating GFP variants with increased fluorescence intensities have been shown to be suitable for BiFC studies in green monkey kidney fibroblast (COS) cells [82].

Another important aspect is to what extent overexpression may produce artefactual results in BiFC assays. Transfection studies with COS cells revealed that high amounts of vector DNA, containing N- and C-terminal YFP sensor peptides respectively, provoked unspecific fluorescence emission [83]. In contrast low amounts of vector resulted in detectable fluorescence only if interacting proteins were fused to the sensor peptides. These data demonstrate that the level of protein expression in BiFC assays has to be carefully controlled to avoid false positive interactions. It is therefore strongly recommended to perform control experiments employing either "empty" vectors or expressing fusion proteins that are not expected to interact with the POIs. In this context it is also worthwhile mentioning that the commonly used BiFC vectors for *in planta *expression generally contain the strong constitutive CaMV 35S promoter [68,69]. It has been demonstrated that CaMV 35S-expressed C- and N-terminal sensor peptides can produce a certain degree of fluorescence even if they are not fused to interacting proteins. This indicates that complementary sensor peptides are capable of a non-assisted interaction (NAI) [80]. However, NAIs are observed only if the complementary sensor peptides are located in the same subcellular compartment. This highlights the fact that appropriate negative controls in BiFC association studies have to be located in the same compartment as the interaction partners under investigation. Development of novel fluorophore derivatives that can be split in less "sticky" halves, either by protein design or *via *random mutagenesis, may diminish NAI-associated difficulties in the future.

Although NAIs pose a problem in interaction studies, they are quite useful to determine the subcellular localization of a protein or a protein domain. In NAI-based localization studies, one sensor peptide is fused to the POI while the complementary sensor peptide is fused to a targeting signal for a suspected subcellular destination (e.g. targeting signal for the nucleus, chloroplasts or mitochondria). If the compartment-targeted sensor peptide produces fluorescence in combination with a protein that is fused to the complementary sensor peptide, one can deduce that the investigated protein localizes to the sensor peptide-targeted compartment. This assay has been recently used to determine the topology of an integral membrane protein [80]. In the future, this approach might become a common procedure to complement GFP-based protein localization studies.

Based on its designation one might expect that BiFC provides a direct measure for bimolecular interactions. However, as for all FCAs that are carried out *in vivo*, it is possible that a third protein mediates the interaction. In this case the observed interaction would be indirect. It has been estimated that fluorescence complementation can occur when fragments are fused to positions that are separated by a distance of approximately 10 nm (100 Å), provided that there is enough flexibility to allow reconstitution of the split YFP fragments [67]. Due to these topological constraints, BiFC will strongly favor the detection of direct protein-protein interactions as opposed to those that occur through complexes. In this context it also needs to be considered that N- or C-terminal halves of the GFP derivative used can be fused to the N- or C-terminal part of the POIs, thus resulting in four different combinations that can theoretically be tested. BiFC-based analysis of interacting proteins has revealed that not all possible combinations of the fusion proteins may give rise to identical results [69], suggesting that each POI should be fused with all possible sensor peptide combinations to ensure fidelity of the experimental outcome.

Perhaps the most exciting application of the BiFC reporter system is the possibility of saturating high-throughput *in planta *interactor screens and thus the replacement of the conventional yeast two-hybrid assay. The envisaged BiFC-based interactor screen of cDNA expression libraries has already been carried out in a suspension cell culture of mammalian COS cells [84]. In brief, a cDNA library was fused to the N-terminal half of GFP while the bait protein was fused to the C-terminal half of GFP. After co-transfection of the bait protein and the prey library, flourescing COS cells were collected by fluorescence-activated cell sorting (FACS), an approach that allows spectral analysis and sorting of 1,000–10,000 cells per second. Expression plasmids were extracted from the collected fluorescing cells and clones encoding putatively interacting proteins further enriched in a second round of co-transfection/cell sorting. Inserts of positive clones were subsequently subjected to DNA sequencing. It remains to be seen whether this screen indeed yielded authentic interactors since the identified candidates have not been studied yet by complementary approaches. To date, BiFC studies in plants have been conducted by either particle bombardment-based or *Agrobacterium*-mediated transient transformation of plant tissues (Table 1). However, in order to accomplish saturating mass screens, plant scientists need to transfer the BiFC assay to suspension cell culture or protoplast systems. In addition, automated analyses in a microtitre plate format or by the aid of a cell sorter (FACS) will be needed to perform such high-throughput BiFC interactor screens *in planta*.

### Beyond conventional FRET and BiFC: studying multiple interactions simultaneously and analyzing interactions with more than two partners

Many proteins potentially have a large number of alternative interaction partners in each cell. Some of these interactions might be mutually exclusive, possibly resulting in competition for shared interaction partners. Interactions between alternative partners in living cells can be studied by a multicolor BiFC assay [85]. This assay is based on the use of fragments of fluorescent proteins with distinct spectral characteristics. Bimolecular complexes formed between these fragments can be visualized using different excitation and emission wavelengths, enabling parallel visualization of multiple interactions in the same cell. Systematic analysis of twelve combinations of different GFP, YFP and CFP sub-fragments resulted in the identification of twelve bimolecular fluorescent complexes with seven distinct spectra [85] that provide an ideal basis for multicolor BiFC. This advancement will not only allow monitoring alternative interaction partners of a POI, but also studying multiple pair wise interactions simultaneously inside the same cell. However, the use of this system is limited to laboratories that have sophisticated detection systems that are capable to discriminate between several GFP derivatives with similar excitation and emission spectra. In contrast to BiFC, it appears that due to inevitable crosstalk between the currently available fluorophores, FRET is confined to a single interaction pair within a particular cell.

Many meaningful biological protein interactions involve polypeptide complexes with more than two interacting proteins. Conventional FRET or BiFC between two components has been unable to shed light on the establishment and/or dynamics of such multi-protein complexes. Recently a three component FRET system based on sensitized emission and DFRAP was described in the context of a three way protein-protein interaction in mammalian cells. ECFP, EYFP and mRFP fused to three different proteins revealed mutually dependent energy transfer between the three fusion proteins in an endosomal compartment [86]. Though careful experimental and theoretical considerations are required to discriminate sequential from parallel energy transfer, this method holds a great promise to characterize three-way interactions during complex signaling processes in plant cells as well.

### Comparison between FRET and BiFC

Both FRET and BiFC generally provide reliable *in planta *protein-protein interaction data. However, as outlined above in detail, both approaches have their individual advantages and disadvantages (Table 2). This is primarily due to the fact that BiFC is based on a gain of fluorescence, while FRET causes a quantitative change in fluorescence. Since FRET-based studies rely on specific detection of spectrally similar fluorophores or even quantification of fluorophore lifetimes, they require sophisticated, expensive instrumentation while BIFC can be measured by standard epifluorescence microscopy equipment [46,53]. Likewise, FRET assays need comprehensive post-imaging data analysis, while this additional step is generally not required for BiFC studies. Since BiFC sensor peptides fluoresce only upon interaction of their fusion partners, it is impossible to visually confirm that both fusion proteins are being made in the absence of an interaction. Thus, in BiFC studies, rather time consuming immunoblot analysis is required to validate expression of the fusion proteins in the absence of interaction. In contrast, FRET sensor peptides are intrinsically fluorescent, which permits detection and quantification of fusion protein levels independently of their interaction status. The irreversibility of the re-established fluorophore complex in BiFC assays is an ambivalent facet: on one hand, this feature enhances sensitivity in determining low-affinity interactions; on the other hand, this attribute may be the cause of false-positive results and also prevents the analysis of dynamic interactions. In this context, false-positive BiFC results may result from high expression levels, while at least some FRET techniques (DFRAP, FLIM) are largely independent of fluorophore concentrations (and thus independent of equimolar and/or physiological expression levels). Finally, BiFC resolves interactions with a high intracellular spatial resolution and is additionally suitable for medium- to high-throughput approaches. In conclusion, the choice between BiFC and FRET depends on the available instrumentation, the skills of the researcher and the experimental requirements. Since BiFC and FRET represent complementary experimental approaches and since in both cases false-negative data may result from trivial causes such as fusion protein stability or unfavorable polypeptide conformation, we generally recommend pursuing both techniques whenever possible.

**Table 2:** Comparison between BiFC and FRET.

	**BiFC**	**FRET**
**Required microscopic equipment**	simple	extensive
**Data analysis and computation required**	-	+
**Concentration dependence**	high	high (channel FRET, FSPIM) or low (DFRAP, FLIM)
**Specific problems**	false positives (possibly due to high expression levels and/or irreversibility)	donor bleed-through (channel FRET, FSPIM), photoconversion, protein mobility (DFRAP)
**Endogenous expression control (i.e. visualization of tagged partners with subcellular resolution)**	-	+
**Monitoring interaction dynamics**	- (fluorophore reconstitution irreversible)	+ (interactions reversible)
**Subcellular resolution of interaction sites**	high	high (FLIM) or low (channel FRET, FSPIM or DFRAP)
**Suitable for tri-molecular interactions**	-	+/-
**Suitable for monitoring multiple distinct interaction pairs inside the same cell ("multicolor")**	+	-
**Suitable for medium to high throughput**	+	-

## Conclusion and outlook

During the past few years, FRET and BiFC have been established as reliable techniques for the analysis of protein-protein interactions in living plant cells. However, one of the obvious disadvantages of both approaches is the fact that they often involve ectopic expression and/or overexpression of the respective fusion proteins (see above and Table 1). This may cause artifacts that could possibly either promote or inhibit particular protein-protein interactions. Thus, fluorophore-based *in planta *protein-protein interaction assays that operate at low, physiological expression levels or even at the single molecule stage are highly desirable. Dual-color Fluorescence cross correlation spectroscopy (FCCS) represents such a method that is based on single molecule detection. This technique involves recognition of two fluorophore-tagged polypeptide species in a sub-femtoliter measurement volume. The two polypeptides are marked with distinct fluorescent labels that can be separately excited and detected. Coincidence of signal fluctuations of both fluorophores in the detection volume indicates co-migration and thus association of the two proteins at the single molecule level [87,88]. Despite the potential power of this method, it has not been extensively applied *in vivo *to date [88] and we do not know of any application in the plant sciences yet. It remains to be seen whether FCCS (or advances of related techniques [89]) will evolve as the next generation of sophisticated *in planta *protein-protein interaction assays.

## Abbreviations

BFP Blue Fluorescent Protein

BiFC Bimolecular Fluorescence Complementation

CaMV Cauliflower Mosaic Virus

CFP Cyan Fluorescent Protein

DFRAP Donor Fluorescence Recovery after Photobleaching

FCA Fragment Complementation Assay

FCCS Fluorescence Cross Correlation Spectroscopy

FLIM Fluorescence Lifetime Imaging

FRET Fluorescence (Förster) Resonance Energy Transfer

FSPIM Fluorescence Spectral Imaging Microscopy

GFP Green Fluorescent Protein

NAI Non-Assisted Interaction

POI Protein of Interest

RFP Red Fluorescent Protein

YFP Yellow Fluorescent Protein

## Competing interests

The author(s) declare that they have no competing interests.

## Authors' contributions

RAB wrote the draft for the FRET section, TL was the main author for the BiFC part. RAB designed schemes depicted in Figures 1, 2, 3 and 5. RP wrote the Abstract, Introduction and Conclusion sections, copy-edited the manuscript, and suggested Figure layouts. All authors have read and agreed on the final version of the manuscript.
